# Supplementary material for: Arctic Ocean virus communities and their seasonality, bipolarity, and prokaryotic associations
Source: Nat Commun. 2025 Jul 11;16:6427. doi: 10.1038/s41467-025-61568-6 (PMC12254263; doi:10.1038/s41467-025-61568-6)
Supplement: Supplementary file 1 — Supplementary Information [file 41467_2025_61568_MOESM1_ESM.pdf]

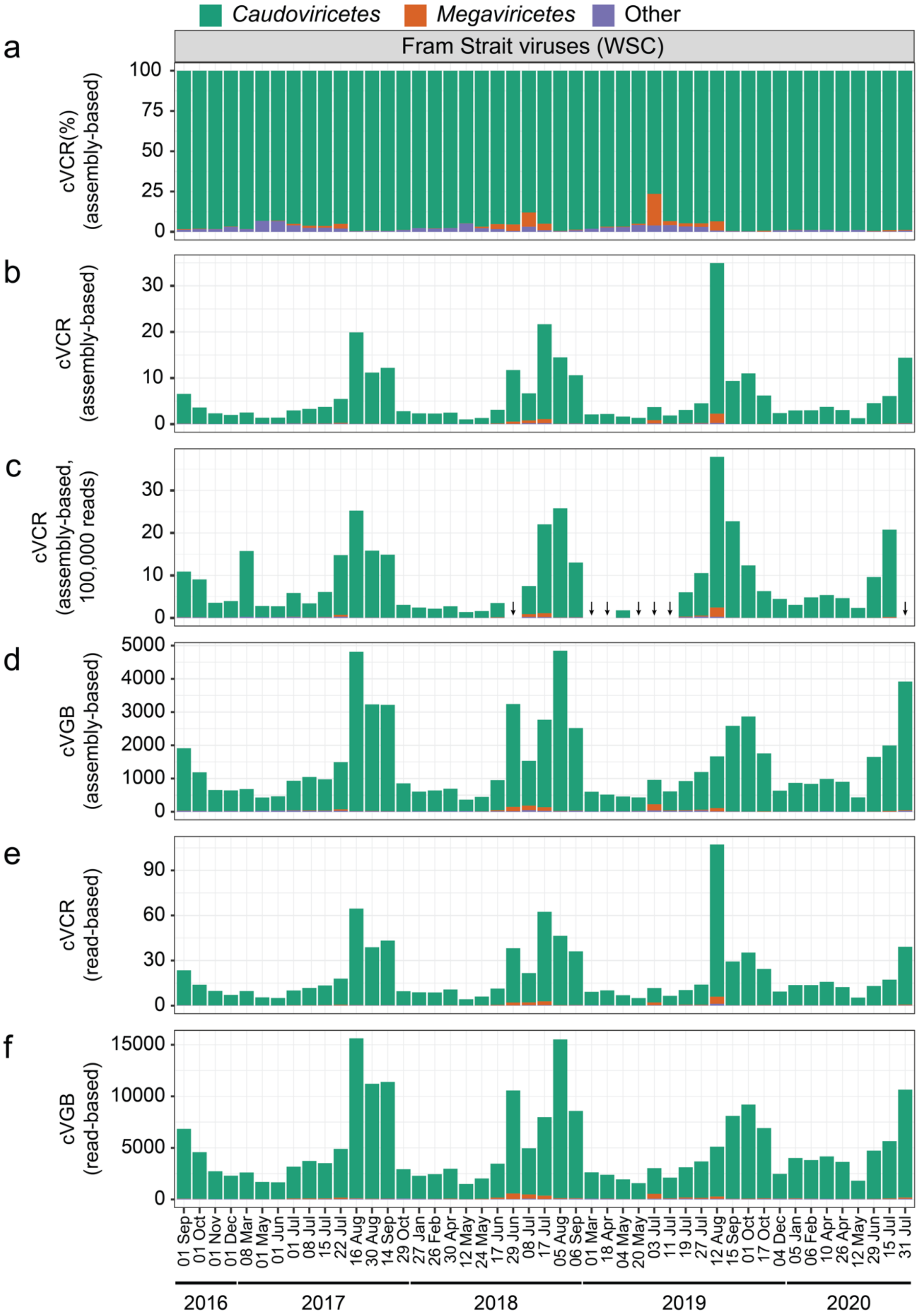

S. Fig. 1. Temporal variation of virus community cVCR and cVGB in the Fram Strait. **a**, proportion of cVCR across viral communities (assembly-based), grouped by taxonomic class. **b**, absolute cVCR values (assembly-based) from viral contigs. **c**, absolute cVCR values (assembly-based) from viral contigs, calculated after subsampling 100,000 reads. Samples with fewer than 100,000 raw reads are marked

with an arrow. **d**, absolute cVGB values (assembly-based) from viral contigs. **e**, absolute cVCR values (read-based) from viral reads. **f**, absolute cVGB values (read-based) from viral reads.

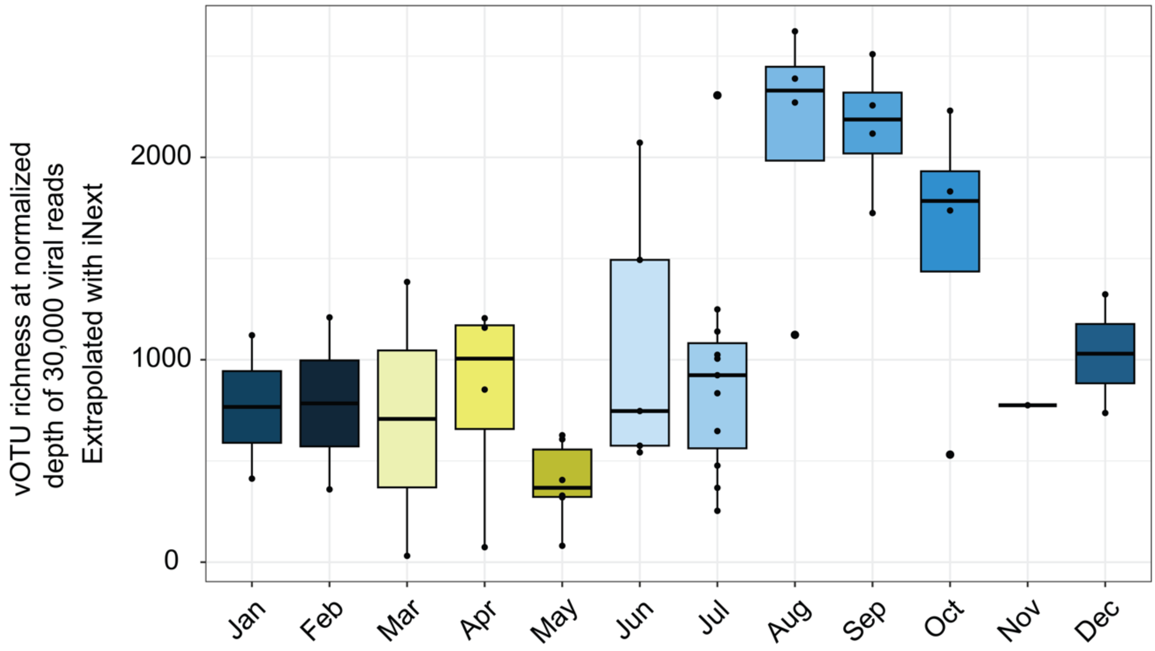

S. Fig 2. Extrapolated richness of vOTUs by month. Boxplots illustrate the richness of the vOTU richness across months as calculated by iNext. Similar to Fig. 2b, the values represent the trajectory of vOTU discovery, but instead of using a linear regression to 1000 rarified reads, iNext which extrapolates to 30,000 reads was used. As in Figure 2, box encompasses the 25th, median, and 75th percentile, and the whiskers capture the minimum and maximum values of richness estimated from samples collected within the same sampling month.

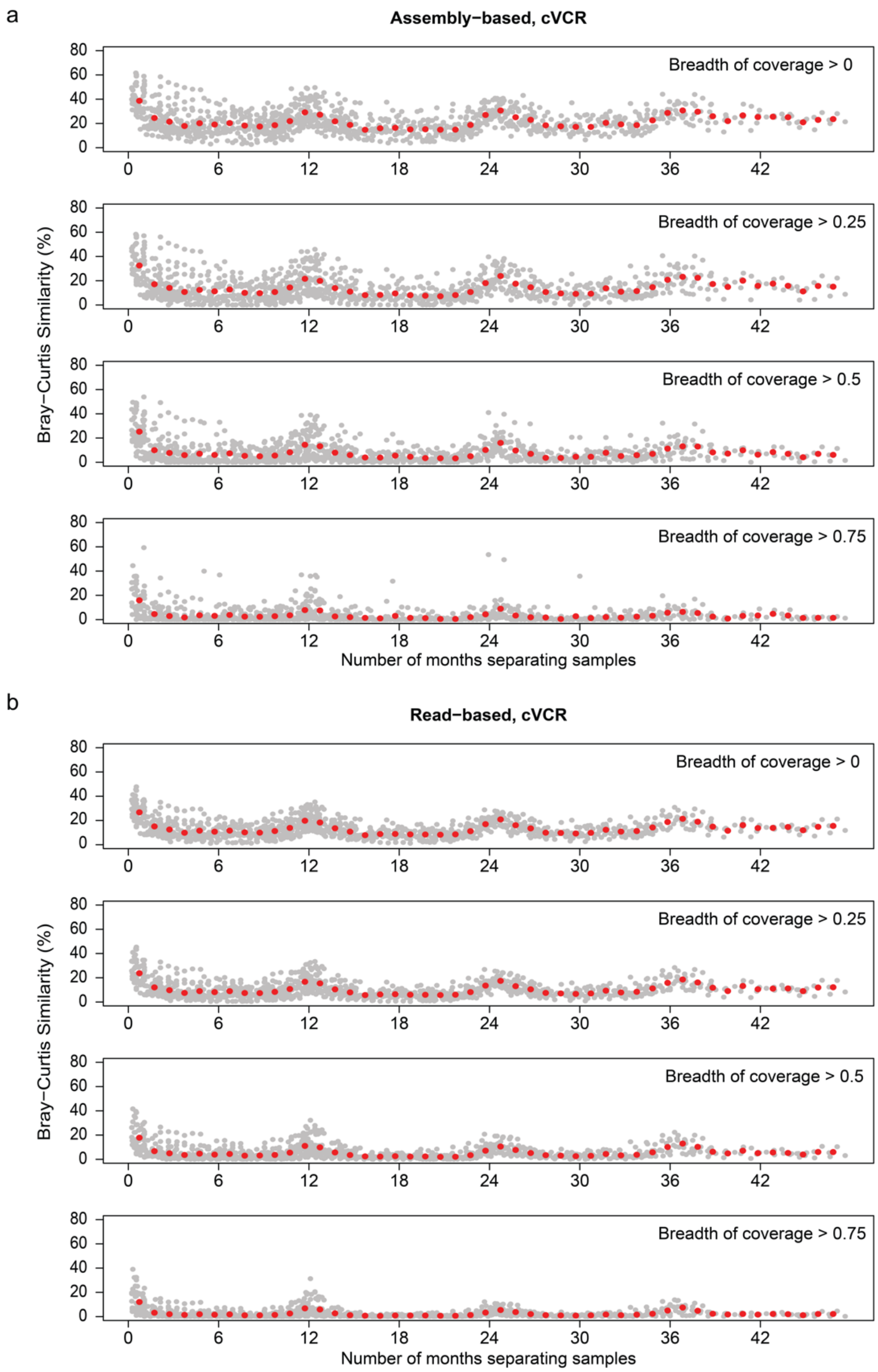

S. Fig. 3. Bray-Curtis similarity of viral communities over time shown for different breadth of coverage cutoffs (>0, >0.25, >0.5, >0.75) using **a**, assembly-based approach, and **b**, read-based approach. Each point represents the similarity (y-axis) between two individual sampling points over time (x-axis). The red points indicate the average similarity for ~monthly intervals.

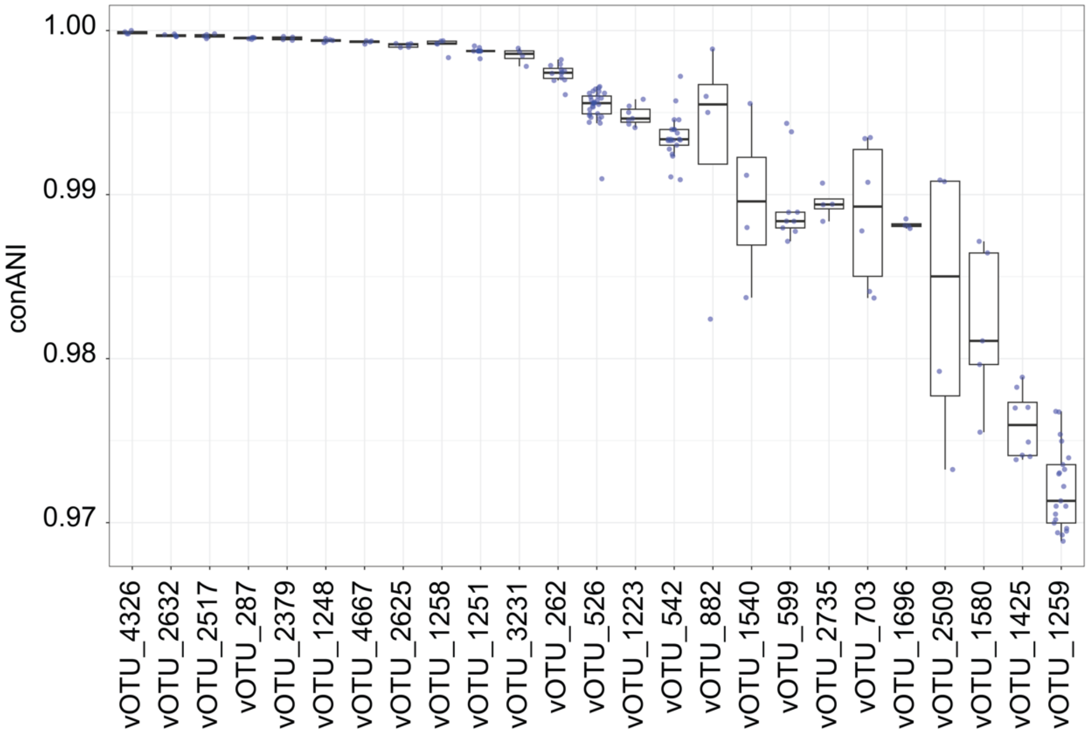

S. Fig 4. Microdiversity in vOTUs. Consensus ANI (conANI) metrics from inStrain are shown for vOTUs exceeding the necessary coverage values three times as described in the Methods. Centre line represents the median. Box limits are the upper quartile and lower quartile. Whiskers represent 1.5 x interquartile range (excluding outliers). All points shown via jitter.

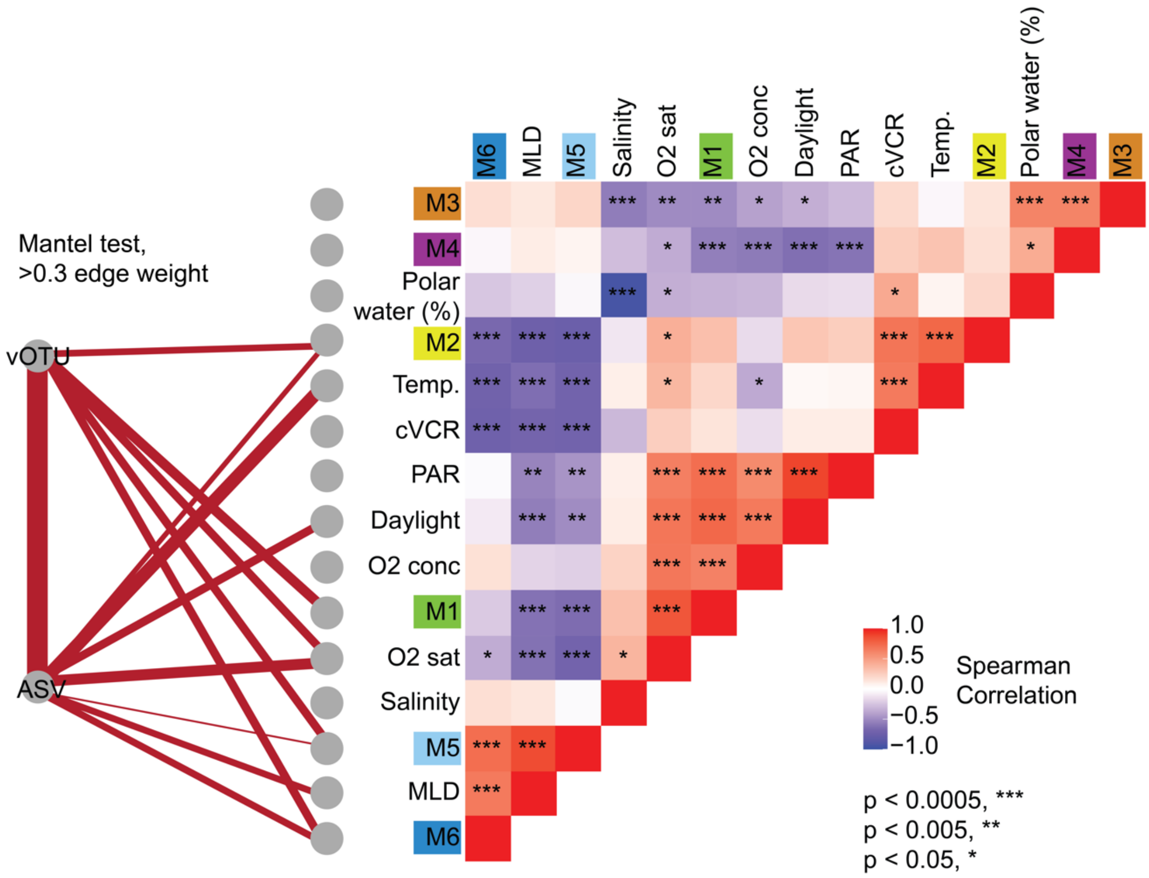

S. Fig. 5. Statistical associations between viral communities, prokaryotic ASVs, and environmental parameters. The network (left) shows Mantel test results, where nodes represent viral communities (vOTUs), prokaryotic communities (ASVs), major modules, and environmental parameters, with edges indicating relationships with Mantel ρ > 0.3. The heatmap (right) displays Spearman correlations. O2 conc, oxygen concentration. O2 sat, oxygen saturation. Temp., temperature.

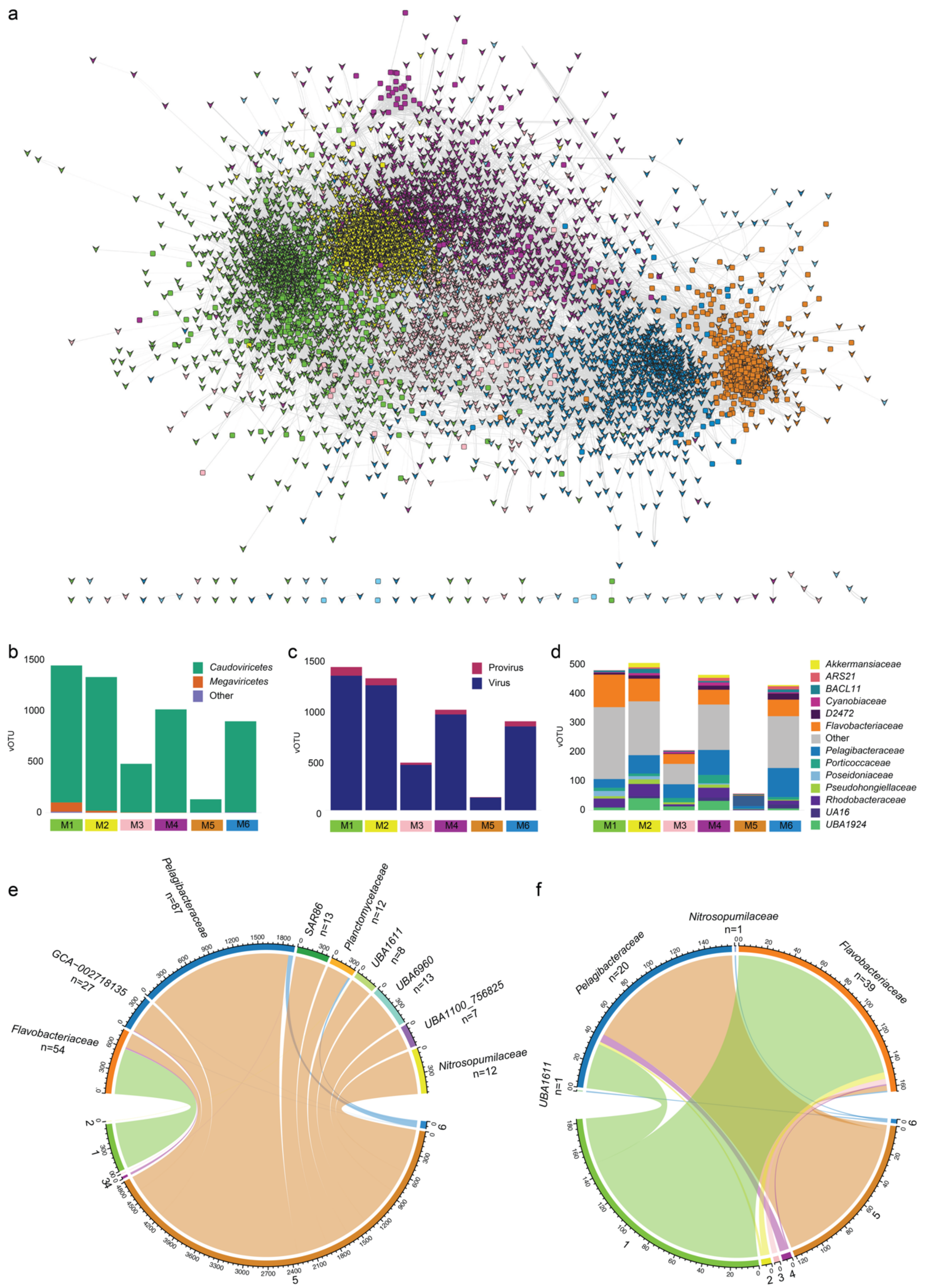

S. Fig. 6. Viral and prokaryotic members within major modules. **a**, Network showing the relationships between vOTUs (arrows) and prokaryotic ASVs (squares). Modules, defined as sets of densely connected nodes, are shown in distinct colors. **b**, Taxonomic composition of vOTUs in each major module. **c**, CheckV prediction for

vOTUs in each major module. **d**, Taxonomy of predicted hosts of vOTUs in each major module. **e**, Chord diagram showing the taxonomic and module distributions of ASVs involved in ASV-to-vOTU correlations. The number of ASVs for each family level is shown around the outside. Only families with at least 250 ASV-to-vOTU correlations are shown. **f**, Chord diagram of the host predictions of vOTU across respective modules. The chord-counts (i.e, around the circle) represent the total number of ASV correlations for each family, while the number of participating vOTUs are shown in text around the outside of the chord diagram.

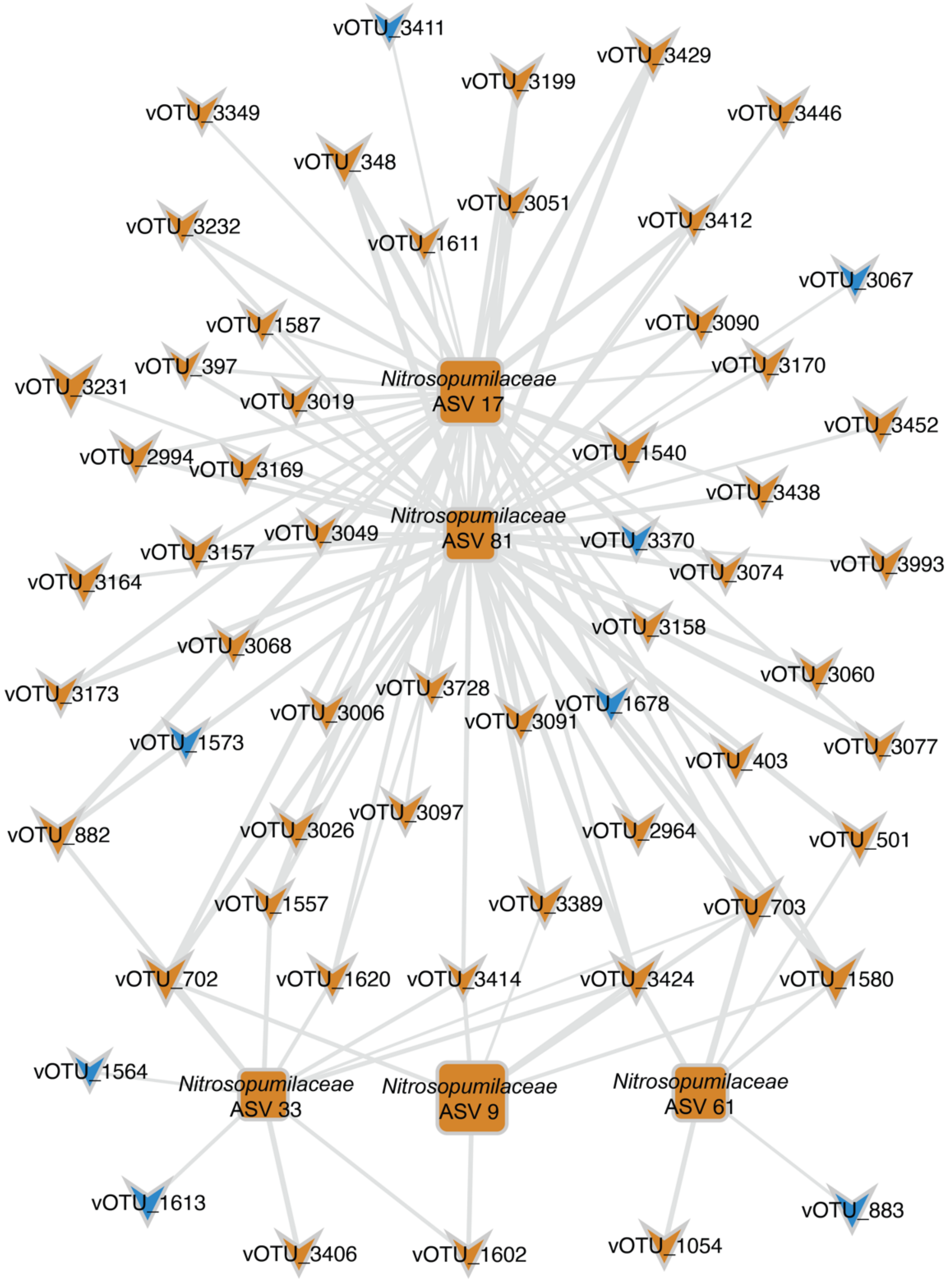

S. Fig. 7. Convergent cross mapping (CCM) network of *Nitrosopumilaceae* ASVs and associated vOTUs (arrows) from modules 5 (orange) and 6 (blue).

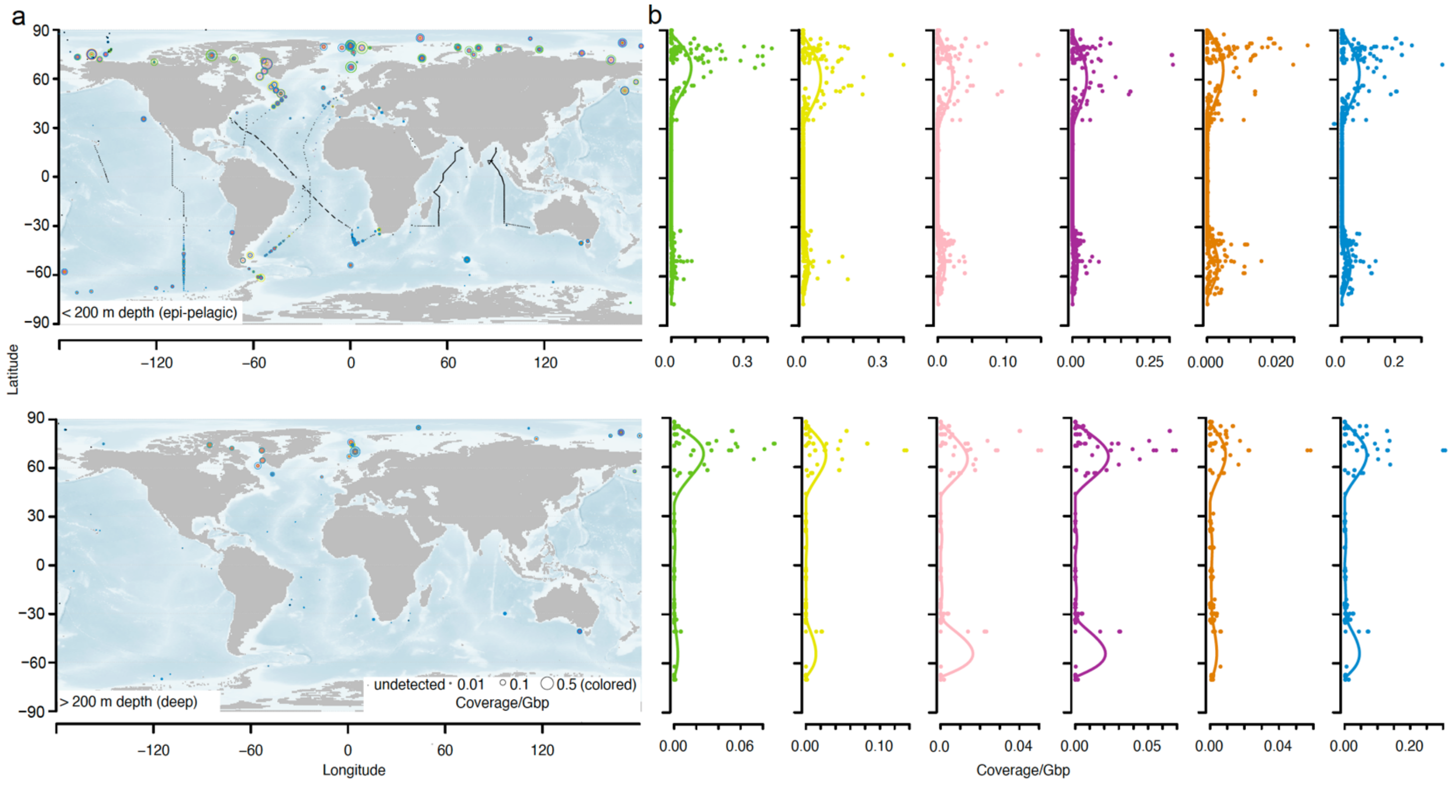

S. Fig. 8. Global distribution of major viral modules in the Fram Strait. Each module is represented by a distinct colour. **a**, Upper map displays vOTUs with mapping data from samples collected above 200 m and bottom from below 200 m. **b**, Coverage/Gbp of each major module plotted by latitude across all samples along with a Generalized Additive Model (GAM) prediction.

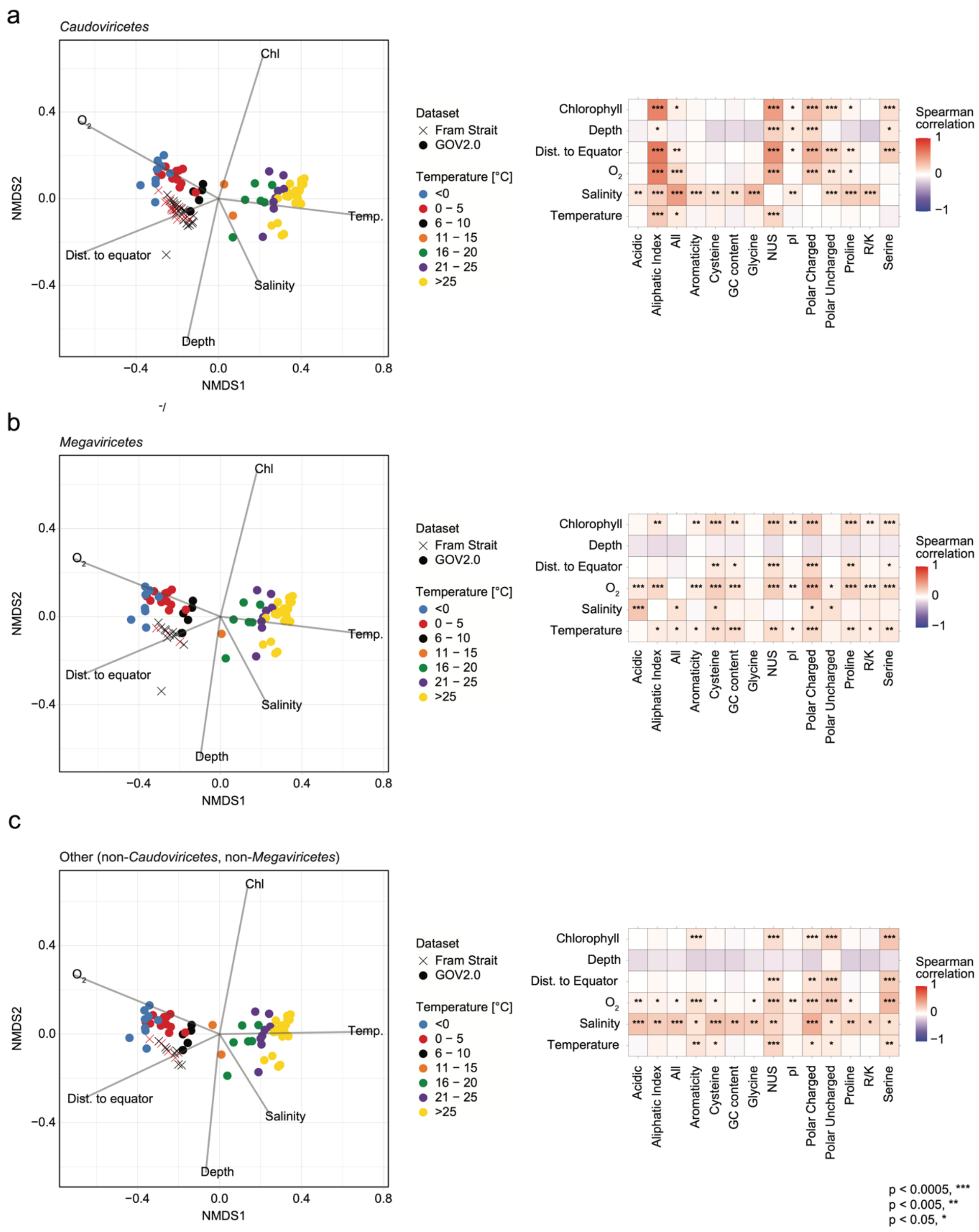

S. Fig. 9. Correlation of amino acid traits to environmental parameters. Like Figure 8, NMDS plots show the relationships between the environmental parameters of the samples. Here samples have been filtered to show the relationships only for samples where **a**, *Caudoviricetes*; **b**, *Megaviricetes*; and **c**, Others (non-*Caudoviricetes*, non-*Megaviricetes*) were detected. Also like Figure 8, heatmap plots (right) illustrate the Spearman correlation coefficients of environmental parameters to amino acid traits, with p-values represented by asterisks as indicated. The Spearman coefficients (two-

sided) were calculated using a Mantel test using pairwise distances of each environmental parameter (Euclidean distance) vs. each amino acid trait (Bray-Curtis distance).

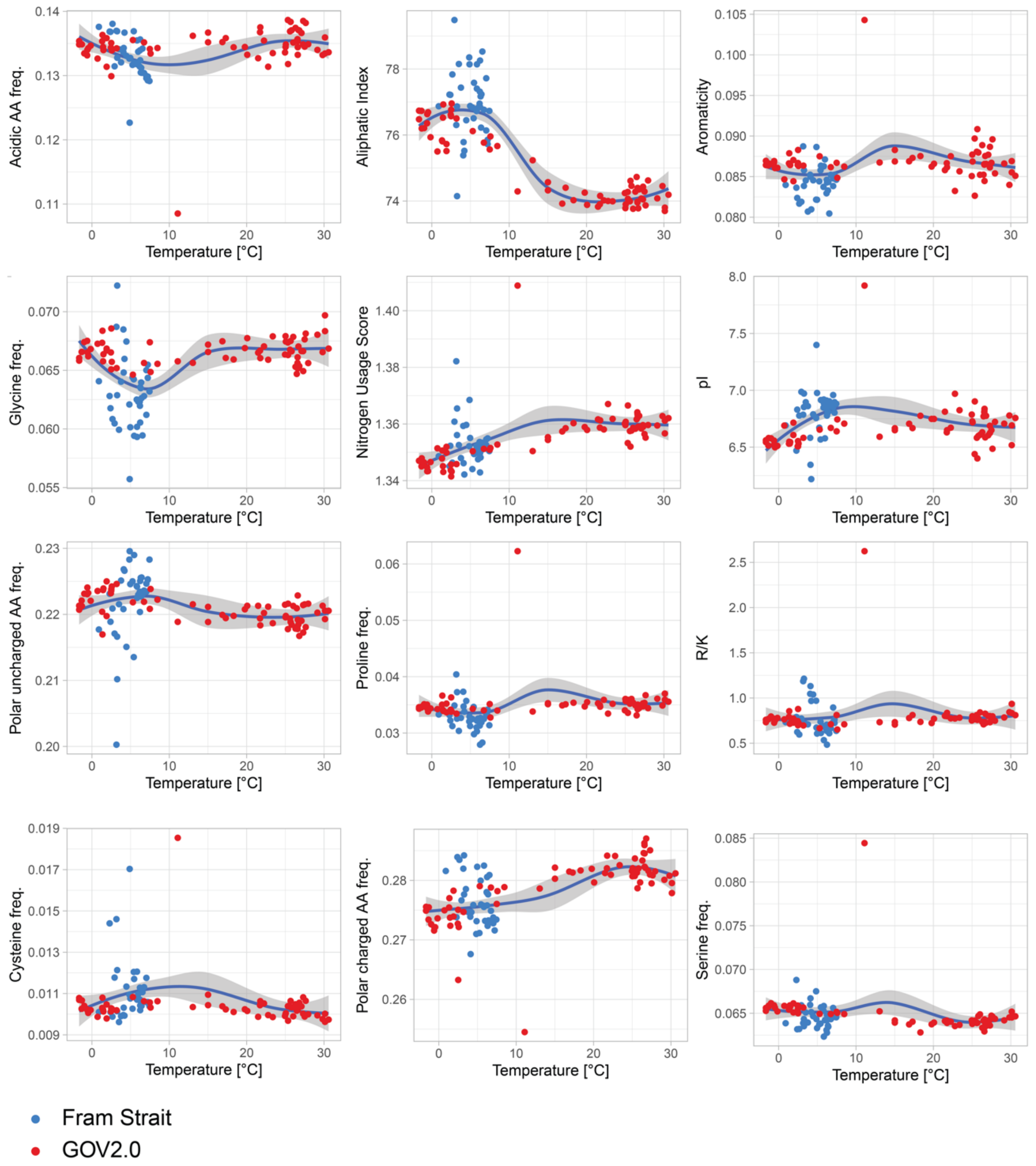

S. Fig. 10. Correlation of protein parameters with temperature. Each panel shows the relationship of temperature and the mean of the specified protein parameters calculated for each sample from this study for both the Fram Strait (blue) and GOV2.0 (red) datasets. The blue line represents a trend line using LOESS curve fitting with a 95% confidence interval (grey).

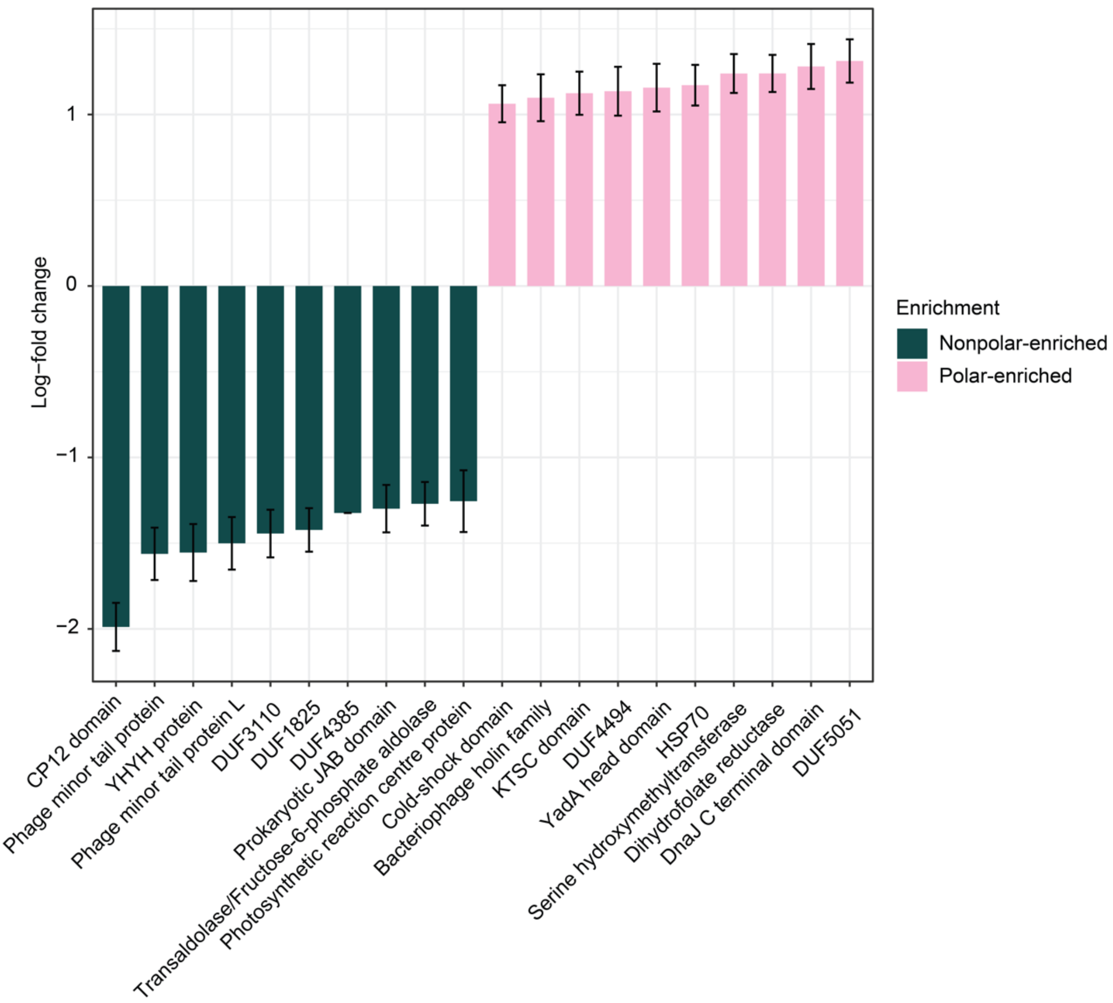

S. Fig. 11. ANCOM-BC results showing protein families that are significantly enriched (top 10) at sampling locations of greater than 60 latitude ($p < 0.001$) or less than 60 latitude. The remaining list with p-values and significance scores as Supplementary Data 8.
