# Supplementary material for: Arctic Ocean virus communities and their seasonality, bipolarity, and prokaryotic associations
Source: Nat Commun. 2025 Jul 11;16:6427. doi: 10.1038/s41467-025-61568-6 (PMC12254263; doi:10.1038/s41467-025-61568-6)
Supplement: Supplementary file 2 — Description of Additional Supplementary Information [file 41467_2025_61568_MOESM2_ESM.pdf]

Description of Additional Supplementary Files

File Name: Supplementary Data 1
Description: Sample information about Fram Strait metagenomes used in the current study. Viral read counts were determined by both mapping reads to vOTUs (Column AM) and by applying viral prediction pipeline at the read-level (Column AN). After removing reads that mapped to vOTUs, cellular read counts and proportions were derived from deep-learning based taxonomy classifier, Tiara v. 1.0.3. In this context, archaea refers to archaeal sequences, bacteria to bacterial sequences, eukarya to eukaryotic sequences, prokarya to prokaryotic sequences that could not be distinguished between archaea and bacteria, and organelle to mitochondrial or plastidial genomes. (Ref: Michał Karlicki, Stanisław Antonowicz, Anna Karnkowska, Tiara: deep learning-based classification system for eukaryotic sequences, Bioinformatics, Volume 38, Issue 2, January 2022, Pages 344–350, https://doi.org/10.1093/bioinformatics/btab672).

File Name: Supplementary Data 2
Description: vOTU information on Fram Strait viruses identified in the current study. The first column for iPHoP prediction contains the combined iPHoP and RaFAH output summarized at the family rank. The second iPHoP prediction column contains iPHoP-only predictions, linked to individual genomes in the custom iPHoP database. Both predictions are outputs from iPHoP.

File Name: Supplementary Data 3
Description: Mantel r statistics for various combinations of biological and environmental parameters. Statistics were determined using Bray-Curtis distances computed with different filtering criteria: all vOTUs, no Megaviricetes vOTUs, no unknown vOTUs, and no Megaviricetes or unknown vOTUs. The explanation for performing multiple sets of Mantel tests was because not all viruses are predicted to be viruses of prokaryotes (and we were only utilizing 16S data, not 18S for eukaryotes), yet it is the most straight-forward and comprehensive to do so. However, we wanted to see also what the correlations were if we excluded likely viruses of eukaryotes. Thus, we also correlated only the Caudoviricetes with the bacterial community, and the other environmental parameters for good measure. These tests showed the differences were negligible, thus we focus our main analysis on the entire virus community compared to the prokaryotic community as well as other parameters, which is column 1 (All vOTUs).

File Name: Supplementary Data 4
Description: Information specifically about the vOTUs that are both correlated with and have host predictions for the same taxon.

File Name: Supplementary Data 5
Description: eLSA results for cyanobacteria and putative cyanophages.

File Name: Supplementary Data 6
Description: Taxonomy and host prediction of vOTUs that correlated with Nitrosopumilaceae ASVs.

File Name: Supplementary Data 7
Description: Project accession information of public data used in mapping Fram Strait viruses to global metagenomes.

File Name: Supplementary Data 8
Description: ANCOM-BC results showing protein families that are significantly enriched at sampling locations of greater than 60 latitude ($p < 0.001$) or less than 60 latitude.
