# Supplementary material for: Arctic Ocean virus communities and their seasonality, bipolarity, and prokaryotic associations
Source: Nat Commun. 2025 Jul 11;16:6427. doi: 10.1038/s41467-025-61568-6 (PMC12254263; doi:10.1038/s41467-025-61568-6)
Supplement: Supplementary file 11 — Reporting Summary [file 41467_2025_61568_MOESM11_ESM.pdf]

nature portfolio

Corresponding author(s): David Needham

Last updated by author(s): 26 May 2025

Reporting Summary

Nature Portfolio wishes to improve the reproducibility of the work that we publish. This form provides structure for consistency and transparency in reporting. For further information on Nature Portfolio policies, see our Editorial Policies and the Editorial Policy Checklist.

Statistics

For all statistical analyses, confirm that the following items are present in the figure legend, table legend, main text, or Methods section.

n/a	Confirmed	
☐	☒	The exact sample size (*n*) for each experimental group/condition, given as a discrete number and unit of measurement
☐	☒	A statement on whether measurements were taken from distinct samples or whether the same sample was measured repeatedly
☐	☒	The statistical test(s) used AND whether they are one- or two-sided<br>*Only common tests should be described solely by name; describe more complex techniques in the Methods section.*
☐	☒	A description of all covariates tested
☐	☒	A description of any assumptions or corrections, such as tests of normality and adjustment for multiple comparisons
☐	☒	A full description of the statistical parameters including central tendency (e.g. means) or other basic estimates (e.g. regression coefficient) AND variation (e.g. standard deviation) or associated estimates of uncertainty (e.g. confidence intervals)
☐	☒	For null hypothesis testing, the test statistic (e.g. *F*, *t*, *r*) with confidence intervals, effect sizes, degrees of freedom and *P* value noted<br>*Give P values as exact values whenever suitable.*
☐	☒	For Bayesian analysis, information on the choice of priors and Markov chain Monte Carlo settings
☒	☐	For hierarchical and complex designs, identification of the appropriate level for tests and full reporting of outcomes
☐	☒	Estimates of effect sizes (e.g. Cohen's *d*, Pearson's *r*), indicating how they were calculated

Our web collection on statistics for biologists contains articles on many of the points above.

Software and code

Policy information about availability of computer code

Data collection	All details regarding collection are presented in the manuscript. Workflows specific to our paper are available via github: https://github.com/alyzzabc/fram_strait_viruses_2016-2020/. Addition information is also available in cited works in particular https://github.com/tpriest0/Fram_Strait_WSC_time_series_2016-2020
Data analysis	Similarly, all software is mentioned in the manuscript, and workflows specific to this manuscript are available https://github.com/alyzzabc/fram_strait_viruses_2016-2020/. Additionally, workflows related to the 16S analysis are available: https://github.com/matthiaswietz/FRAM_eDNA). The softwares and version utilized in the data analysis, as listed in the methods are as follows: hifiasm-meta v.0.13-r308, VirSorter2 v.2.2.3, CheckV v.1.0.1, DeepMicroClass v.1.0.3, cd-hit v.4.8.1, geNomad v.1.8.1, vConTACT3 v.3.0.0b65, iPHoP v.1.3.3, minimap2 v.2.28-r1209, Tiara v.1.0.3, R v.4.3.1, VPF-class v.0.1.2, inStrain v.1.9.0, Prodigal v.2.6.3, BBTools v.39.01, iNEXT v.3.0.1

For manuscripts utilizing custom algorithms or software that are central to the research but not yet described in published literature, software must be made available to editors and reviewers. We strongly encourage code deposition in a community repository (e.g. GitHub). See the Nature Portfolio guidelines for submitting code & software for further information.

Data

Policy information about availability of data

All manuscripts must include a data availability statement. This statement should provide the following information, where applicable:

- Accession codes, unique identifiers, or web links for publicly available datasets
- A description of any restrictions on data availability
- For clinical datasets or third party data, please ensure that the statement adheres to our policy

Raw metagenomic reads are available under ENA BioProjects PRJEB67368 (WSC) [https://www.ncbi.nlm.nih.gov/bioproject/PRJEB67368/] and PRJEB52171 (EGC) [https://www.ncbi.nlm.nih.gov/bioproject/?term=PRJEB52171]. 16S rRNA amplicon reads are available under PRJEB43890 (2016-2017) [https://www.ncbi.nlm.nih.gov/bioproject/?term=PRJEB43890], PRJEB43889 (2017-2018) [https://www.ncbi.nlm.nih.gov/bioproject/?term=PRJEB43889], PRJEB67813 (2018-2019) [https://www.ncbi.nlm.nih.gov/bioproject/?term=PRJEB67813], and PRJEB66202 (2019-2020) [https://www.ncbi.nlm.nih.gov/bioproject/?term=PRJEB66202]. Physicochemical parameters are available at PANGAEA under https://doi.pangaea.de/10.1594/PANGAEA.904565 (2016-2017), https://doi.pangaea.de/10.1594/PANGAEA.904534 (2017-2018), https://doi.pangaea.de/10.1594/PANGAEA.941126 (2018-2019), and https://doi.pangaea.de/10.1594/PANGAEA.946508 (2019-2020). vOTU contigs and network files are available via figshare DOIs https://doi.org/10.6084/m9.figshare.28045856 and https://doi.org/10.6084/m9.figshare.28045826, respectively. psbA alignment, trimmed alignment, treefile, and labels for cyanophage references are available via figshare (DOI https://doi.org/10.6084/m9.figshare.28398173).

Research involving human participants, their data, or biological material

Policy information about studies with human participants or human data. See also policy information about sex, gender (identity/presentation), and sexual orientation and race, ethnicity and racism.

Reporting on sex and gender	NA
Reporting on race, ethnicity, or other socially relevant groupings	NA
Population characteristics	NA
Recruitment	NA
Ethics oversight	NA

Note that full information on the approval of the study protocol must also be provided in the manuscript.

Field-specific reporting

Please select the one below that is the best fit for your research. If you are not sure, read the appropriate sections before making your selection.

☐ Life sciences ☐ Behavioural & social sciences ☒ Ecological, evolutionary & environmental sciences

For a reference copy of the document with all sections, see nature.com/documents/nr-reporting-summary-flat.pdf

Ecological, evolutionary & environmental sciences study design

All studies must disclose on these points even when the disclosure is negative.

Study description	Time-series study at a fixed marine location, collecting environmental samples in consistent intervals over four years. note this study is a novel follow-up study based on a published dataset. Please see: https://github.com/tpriest0/Fram_Strait_WSC_time_series_2016-2020. Therefore, many of the descriptions of study design here repeat those from the reporting summary associated with that study.
Research sample	Seawater (collected with autonomous samplers) and physicochemical data (measured by in-situ sensors) on moorings.
Sampling strategy	Monthly to biweekly sampling of seawater. Continous physicochemical measurements via sensors.
Data collection	Data collected and evaluated by co-authors of this paper.
Timing and spatial scale	August 2016 until August 2020
Data exclusions	no data were excluded
Reproducibility	Code is available for workflows within the github: https://github.com/alyzzabc/fram_strait_viruses_2016-2020/
Randomization	Not strictly applicable, details available in MS for statistics.

Blinding	time-series samplings using remote autonomous devices in pre-programmed intervals. Continuous data acquisition.

Did the study involve field work? [x] Yes [ ] No

Field work, collection and transport

Field conditions	marine sampling; at ~30m depth using moored autonomous devices
Location	Fram Strait, open Arctic Ocean (80°N 7°E)
Access & import/export	Deployment/recovery of instruments and samples every summer; shipboard transfer back to home institution. No permits required.
Disturbance	Minimal disturbance, moored instruments fully recovered. No noise or other pollution during mooring operation

Reporting for specific materials, systems and methods

We require information from authors about some types of materials, experimental systems and methods used in many studies. Here, indicate whether each material, system or method listed is relevant to your study. If you are not sure if a list item applies to your research, read the appropriate section before selecting a response.

Materials & experimental systems

n/a	Involved in the study
[x]	[ ] Antibodies
[x]	[ ] Eukaryotic cell lines
[x]	[ ] Palaeontology and archaeology
[x]	[ ] Animals and other organisms
[x]	[ ] Clinical data
[x]	[ ] Dual use research of concern
[x]	[ ] Plants

Methods

n/a	Involved in the study
[x]	[ ] ChIP-seq
[x]	[ ] Flow cytometry
[x]	[ ] MRI-based neuroimaging

Plants

Seed stocks	NA
Novel plant genotypes	NA
Authentication	NA
